# Supplementary material for: End Sequence Analysis Toolkit (ESAT) expands the extractable information from single-cell RNA-seq data
Source: Genome Res. 2016 Oct;26(10):1397–410. doi: 10.1101/gr.207902.116 (PMC5052061; doi:10.1101/gr.207902.116)
Supplement: Supplemental Material [file supp_gr.207902.116_Supp_Materials.tar.gz › ESAT-esat_v0.1/doc/html/classumms_1_1core_1_1readers_1_1_paired_end_reader-members.html]

ESAT: Member List


ESAT


- Main Page
- Related Pages
- Packages
- Classes
- Files

- Class List
- Class Index
- Class Hierarchy
- Class Members

All Classes Namespaces Files Functions Variables Enumerator Pages

- umms
- core
- readers
- PairedEndReader

umms.core.readers.PairedEndReader Member List

This is the complete list of members for umms.core.readers.PairedEndReader, including all inherited members.

|  |  |  |
| --- | --- | --- |
| close() | umms.core.readers.PairedEndReader |  |
| getAlignmentType(SAMFileHeader header) | umms.core.readers.PairedEndReader | static |
| getAlignmentType() | umms.core.readers.PairedEndReader |  |
| getHeader() | umms.core.readers.PairedEndReader |  |
| getOrCreatePairedEndFile(String fileToCheck, TranscriptionRead txnRead) | umms.core.readers.PairedEndReader | static |
| getPairedEndFile(String fileToCheck) | umms.core.readers.PairedEndReader | static |
| getRefSequenceLengths(String chr) | umms.core.readers.PairedEndReader |  |
| getRefSequenceLengths() | umms.core.readers.PairedEndReader |  |
| getSequenceNames() | umms.core.readers.PairedEndReader |  |
| hasIndex() | umms.core.readers.PairedEndReader |  |
| isPairedEndFormat(String fileToCheck) | umms.core.readers.PairedEndReader | static |
| iterator() | umms.core.readers.PairedEndReader |  |
| main(String[] args) | umms.core.readers.PairedEndReader | static |
| PairedEndReader(File bam) | umms.core.readers.PairedEndReader |  |
| PairedEndReader(File bam, TranscriptionRead read) | umms.core.readers.PairedEndReader |  |
| PairedEndReader(File bam, TranscriptionRead read, boolean fra) | umms.core.readers.PairedEndReader |  |
| query(Annotation a, boolean contained) | umms.core.readers.PairedEndReader |  |
| setFragmentFlag(boolean fra) | umms.core.readers.PairedEndReader |  |


---

Generated on Fri Aug 15 2014 15:11:14 for ESAT by  

 1.8.7
